# Artificial intelligence achieves easy-to-adapt nonlinear global temperature reconstructions using minimal local data

**DOI:** 10.1038/s43247-023-00872-9

**Published:** 2023-06-16

**Authors:** Martin Wegmann, Fernando Jaume-Santero

**Affiliations:** 1 Institute of Geography, University of Bern, Bern, Switzerland; 2 Oeschger Centre for Climate Change Research, University of Bern, Bern, Switzerland; 3 Department of Radiology and Medical Informatics, University of Geneva, Geneva, Switzerland; 4 Geneva School of Business Administration, University of Applied Sciences and Arts of Western Switzerland, Carouge, Switzerland

**Keywords:** Palaeoclimate, Atmospheric dynamics, Climate and Earth system modelling

## Abstract

Understanding monthly-to-annual climate variability is essential for adapting to future climate extremes. Key ways to do this are through analysing climate field reconstructions and reanalyses. However, producing such reconstructions can be limited by high production costs, unrealistic linearity assumptions, or uneven distribution of local climate records. Here, we present a machine learning-based non-linear climate variability reconstruction method using a Recurrent Neural Network that is able to learn from existing model outputs and reanalysis data. As a proof-of-concept, we reconstructed more than 400 years of global, monthly temperature anomalies based on sparse, realistically distributed pseudo-station data and show the impact of different training data sets. Our reconstructions show realistic temperature patterns and magnitude reproduction costing about 1 hour on a middle-class laptop. We highlight the method’s capability in terms of mean statistics compared to more established methods and find that it is also suited to reconstruct specific climate events. This approach can easily be adapted for a wide range of regions, periods and variables.

## Abstract (web summary)

A fast and robust solution for global temperature reconstructions can be obtained without the need for training on local station data by using a recurrent neural network machine learning approach that utilises sparse, realistically distributed pseudo-station data.

## Introduction

As global warming remains pervasive, extreme climate and weather events have been highlighted as high-impact threats to our society^1,2^. Fueled by Earth’s energy imbalance, climate variability is projected to increase non-linearly in response to anthropogenic global warming, a process governing the occurrence and distribution of extreme climate events^3–7^. Unfortunately, state-of-the-art General Circulation Models (GCM) lack the ability to represent the correct magnitude of variability in crucial parts of the climate system^8,9^. Gaining more insights into a realistic range of climate variability is therefore of utmost importance.

To investigate realistic climate variability across different temporal and spatial scales with diverse climate background conditions, long, global time series are required. However, the further back in time one goes, the fewer climate and weather observations are available, creating a problem of data scarcity. Near-surface temperature, the variable with the longest records, was initially recorded in just a handful of European cities and has been surveyed since the late 18^th^ century^10–12^. Other variables and southern hemispheric measurements are considerably scarcer. Indeed, to go further back in time, it is necessary to utilize sets of indirect proxy measurements from paleoclimate archives. Compared to observations, climate proxies from paleoclimate archives suffer from a substantially reduced temporal resolution and a significant increase in noise. Moreover, most of the available documentary and proxy data record summer or growing season climate, whereas winter climate is usually underrepresented^13^.

As such, the climate community has made a collaborative effort to investigate tools for temporal and spatial reconstructions of climate and weather data, including methods like kriging, principal component (regression) analysis and Bayesian algorithms. The results of these efforts include high spatial and temporal resolution data sets, mostly focused on specific regions rather than global coverage^14–16^. Climate reconstructions can further be improved by optimizing the input data location using meta-heuristic methodologies such as genetic and evolutionary algorithms^17–19^. Nevertheless, most climate reconstructions today rely on stationarity within and between climate records and often operate on the assumption of linearity within time and space. Climate reanalyses are another possible way to generate spatially resolved climate reconstructions^20–24^. While they offer a four-dimensional data set to explore high-resolution climate variability for a wide range of variables, they are also constrained by the availability of input data (e.g. stations, documentary data, proxy data). It should be noted that, while the assimilation scheme itself can be straightforward, most climate reanalyses require an expensive set of GCM outputs for the background climate state and co-variance matrix. This prerequisite makes the process of generating a climate reanalysis rather expensive, since large amounts of data need to be generated and stored.

Recently, rapid progress was made in the implementation of artificial intelligence tools for climate science^25–27^. In particular, deep learning tools show promise in extracting features of interest out of gridded data, forecasting time series and representing physical systems^28–30^. So far, these deep learning algorithms provide a good compromise between skill and costs, while dealing well with the non-linear characteristics of the data at hand. Their application in climate information reconstruction has been limited to examining gridded data^31,32^ or time series reconstructions^33,34^.

Here, we present a novel approach based on a simple recurrent neural network that reconstructs global fields from sparse local data. Our main goal focuses on proving the effectiveness of basic recurrent neural networks in generating fast robust climate reconstructions using minimal computational power. As such, we stayed extremely conservative in both the spatial and temporal availability of data and therefore operated on very small sample sizes for a deep learning approach. Although temperature anomalies are reconstructed in this study, our method is flexible and can be applied to reconstruct multiple variables from differing local coordinates and input data types. In support of the United Nations Sustainable Development Goals^35,36^, the whole reconstruction process of training and generating more than 4800 months of global temperature anomalies takes just a few minutes on an averaged-priced laptop, making climate research more accessible and energy efficient.

## Results and discussion

**Fig. 1:**
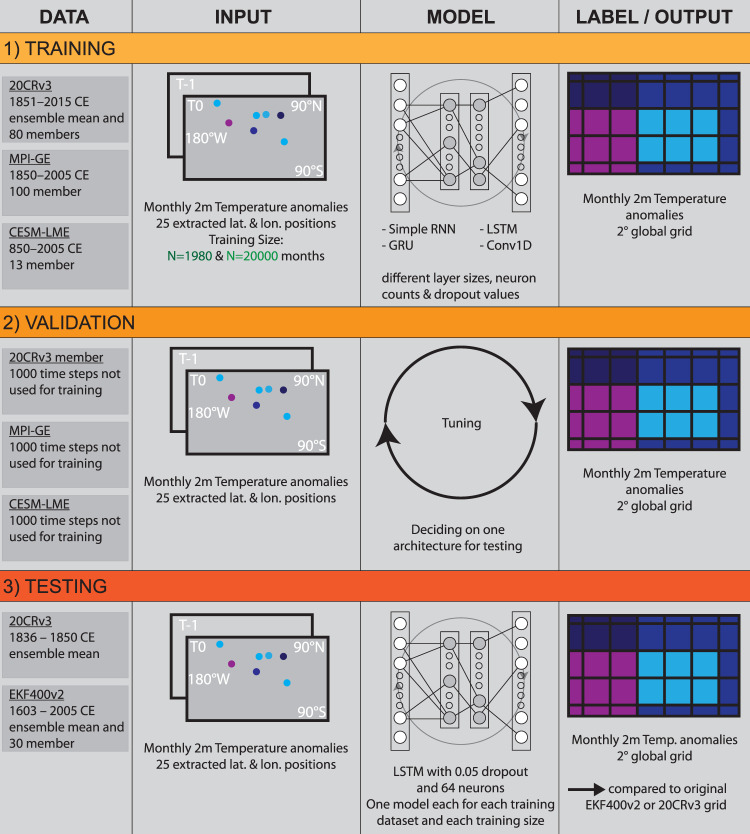
Concept of the reconstruction process. As training data set we use monthly 2m temperature anomalies with respect to the period 1951–1980 CE from three different gridded products, one reanalysis and two coupled climate models. We extract the nearest neighbour information from 25 locations, which are then used to reconstruct the global grid. For validation we use 1000 time steps that are not used for training. Based on the validation results, we choose one architecture out of 140 for the testing phase, where we use 20CRv3 and EKF400v2 data for the time series of the 25 locations in order to reconstruct the 20CRv3 and EKF400v2 fields. These reconstructions are then compared to the actual 20CRv3 or EKF400v2 product.

Figure 1 highlights the workflow of our approach. We use monthly near-surface temperature data from three different gridded climate data sets as training data for our global reconstruction: The National Oceanic and Atmospheric Administration (NOAA) 20^th^ Century reanalysis Version 3 (20CRv3)^22^ (1836-2015 CE, 1851-2015 CE used for the training), the Max Planck Institute for Meteorology Grand Ensemble (MPI-GE) GCM^37^ (1851-2005 CE), as well as, the National Center for Atmospheric Research Community Earth System Model Last Millennium Ensemble (CESM-LME)^38^ (850-2005 CE). We do so in order to understand the impact that training data can have on our approach and to highlight strength and weaknesses of this often-used training data in the climate science space. Furthermore, it helps us understand the amount and variability of data needed for the training. The three datasets used have enough dissimilarities in origin, idea and time frame covered in order to see some meaningful differences. 20CRv3 only assimilates surface pressure data and uses sea surface temperature (SST) and sea ice reconstructions as boundary conditions, whereas MPI-GE and CESM-LME are free running models with transient forcing. All three data sets consist of multiple ensemble members. For most of our study, we focus on the ensemble mean of 20CRv3 since this is the information most users will use as well as to highlight the capabilities of small training sizes for this reconstruction procedure. For all training data sets we calculate monthly temperature anomalies with respect to the period 1951–1980 CE. We utilized these three data sets because they are commonly used in the climate science community, are fully open-access and cover climate information and variables close to the reconstruction goal. Moreover, they are also independent allowing for the study of significant differences on the final reconstructions.

In each of the gridded products, 25 pseudo-station locations chosen based on a realistic distribution of historical meteorological station data. All locations are situated in the Northern Hemisphere, with the majority in Western Europe. Using a nearest neighbour approach, we then extract the grid temperature data for each location in the specific training data set, resulting in 25 monthly near-surface temperature anomaly time series.

Overall we investigated 20 different models using three different data sets and two different training sizes for this study. The models used were the simple Recurrent Neural Network (RNN), Long-short Term Memory models (LSTM), Gated Recurrent Unit models (GRU) and 1-dimensional Convolutional Neural Networks (CNN). Each of these models were then executed with a variety of dropout and layer architectures. Summed up over all these different contributors, we analysed 140 different deep-learning models for this climate reconstruction task.

For the RNN models, we found that most models overfit for the small training size of *N* = 1980 no matter the training data set at hand. In order to avoid overfitting, dropout values would need to be at least 20%. Recurrent dropout will further reduce the chance for overfitting, but we see no additional benefit from recurrent dropout in the RNN-type models. Increasing the training sample size to *N* = 20,000 will drastically reduce the chance for overfitting and in most cases a dropout of just 5% is enough to prevent any kind of overfitting. We performed a small sensitivity test with the MPI-GE training set and found that a training size of *N* = 10,000 with a dropout of 5% is enough to avoid overfitting. A training size of *N* = 5000 still needs a dropout of 20% to do so.

Overfitting is just one metric to assess the usefulness of a model. In terms of the lowest validation loss, we found that very simple models show the best results. On average over 140 models, we found that one layer GRU and LSTM models with 32 or 64 neurons for smaller training sizes and up to 256 neurons for larger sample sizes beat models with more neurons, more layers or convolutional structures. In cases of large training sizes, a second layer to the LSTM did not show worse results, but due to increased complexity showed substantially higher computational costs. Moreover, dropout rates needed to be slightly higher in two layer models to avoid overfitting. During the training phase, we could not identify a distinct preference of models for specific training data sets.

In the evaluation phase of our study we let the trained models reconstruct 1000 unseen time steps of the training data set. We then assessed their skill for the reconstruction using mean squared error (MSE) and Pearson correlation coefficient (R) metrics, common metrics for regression problems. Due to the fact that the 1000 time steps used for evaluation are still part of a population with similar characteristics, the best MSE scores were achieved by simpler models that would generally overfit, namely one layer RNN, GRU and LSTM models with no dropout. However, adding 5 or 10% of dropout to these models still yields very performant MSE values. For models trained on *N* = 20,000 samples, more neurons seem to be appropriate, as can be expected. The only training data for which the CNNs showed an acceptable performance was the 20CRv3 multi-member data set. All other data sets preferred any of the RNN architectures.

For the correlation evaluation, a virtually identical outcome emerges. Simple GRU and LSTM models dominate the performance assessment, small dropout rates have little impact on that performance and models with larger training sizes benefit from more neurons. The robustness of the simple GRU and LSTM architectures for this task was also highlighted by an experiment for which we reduced the time dependence of the prediction in the 20CRv3 data set. We randomized parts of the training data set, reducing the skill that can be learned from the previous time step. In that case, one layer, 64-neuron GRU and LSTM models still outperformed the rest of the models. Naturally, chances of overfitting were reduced in that case and validation losses increased.

To compare the deep learning climate reconstructions with a more established, linear statistical tool, we created a Principal Component Regression (PCR) reconstruction^39,40^ trained with the MPI-GE data set. The PCR training sample size is *N* = 20,000. Compared to the deep learning models, the PCR needs a calibration period which is a time range where pseudo-location information (i.e., testing data) and training data overlap. As the testing data set spans 1602–2003 CE and the MPI-GE data spans 1850–2005 CE, the years in common are 1850–2003, which were chosen as the calibration period for the linear regression. As such we will evaluate the reconstruction for the full period 1602–2003, for months outside the calibration period (1602–1849) and just for the calibration period (1850–2003). Note that the deep learning methods do not require such a calibration period and can be trained purely on an independent data set. That said, due to the fact that the calibration of the PCR substantially removes the bias for the calibration period, we might expect a higher performance of the PCR compared to the deep learning models in those years. We refrain from performing the PCR for all training sizes and training data sets since our point here is to just provide a reference point for the interested reader rather than creating as many reconstructions as possible.

As we move on to the testing phase of our study, we continue with only one of the performant model architectures in order to highlight the impact of the different training data sets. Out of 140 models, we focus on the output of an LSTM architecture model with 64 neurons and a dropout of 5% to compare our reconstructions with the independent test data sets. That said, as this study is designed as proof-of-concept, the reader can adapt this architecture easily for the task and training sample size at hand. Our goal here is not to tune one model on one data set to produce the most specific performance, but to show a range of outcomes possible for this reconstruction task.

For testing, we extract 2m temperature data from the 25 locations in the updated global atmospheric paleo-reanalysis for the last 400 years (EKF400v2)^24^. The task of the trained neural network and the PCR is to reconstruct global temperature anomaly fields for 4824 months covering 1602–2003 CE based solely on the 25 local temperature time series. The reconstruction created by the MPI-GE trained LSTM is henceforth called MPI-GE-REC, same for the 20CRv3 and CESM-LME trained reconstructions.

By using temperature anomalies rather than absolute temperature values no information is given about seasonality. The difficulty for the neural network is thus increased as it must learn the physical relationships within the spatial domain. Given this a-priori setup, we expect a higher skill in boreal winter temperature anomaly reconstruction due to stronger Northern Hemisphere planetary wave interactions in that season^41^. We further expect boreal summer temperature anomalies to be better represented in 20CRv3 than in the coupled climate models due to the assimilated data. With the impact of large-scale circulation prevailing in winter, the different reconstructions should perform rather similarly in boreal winter and differently for boreal summer. We utilize anomaly correlation, mean temperature biases and ability to reconstruct patterns of variability as assessments for the skill of the reconstruction. Since we use EKF400v2 as baseline comparison data set, it is noteworthy to mention that this paleo-reanalysis is an imperfect data set in and of itself. Differences between our reconstruction and EKF400v2 must thus be seen as objective differences. We provide context and explanations for stark dissimilarities whenever possible. Moreover, where applicable, we compare the RNN reconstructions to completely independent products or time intervals. Details of the reanalysis and climate models are described in the Methods section.

### Reconstructed temporal and spatial variability

**Fig. 2:**
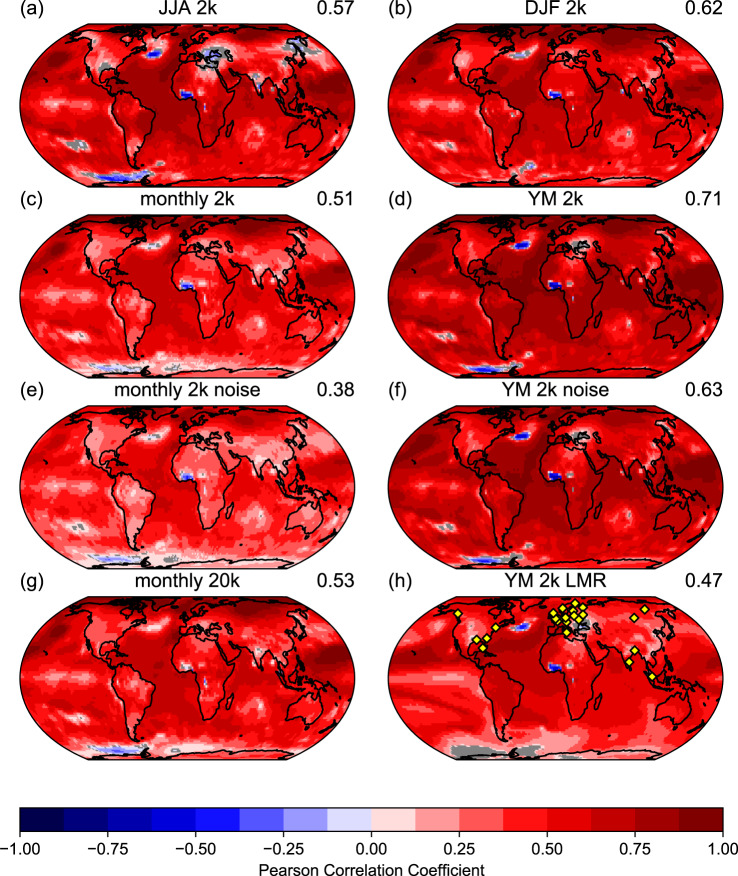
Global distribution of reconstruction performance. Maps of Pearson correlation coefficients between EKF400v2 ensemble mean and the MPI-GE-REC (*N* = 1980 training months) temperature anomaly reconstruction for the period 1602–2003 CE. **a** Correlation coefficients for boreal summer (JJA) seasons (*N* = 402). **b** Correlation coefficients for boreal winter (DJF) seasons (*N* = 401). **c** Correlation coefficients for all months (*N* = 4824). **d** Correlation coefficients for yearly means (*N* = 402). **e** as **d** but instead of extracting pseudo-station data from the EKF400v2 ensemble mean, the pseudo-station data is taken from 30 individual EKF400v2 member, computing 30 global reconstructions which are averaged to achieve a reconstruction ensemble mean. This ensemble mean is then correlated with the EKF400v2 ensemble mean (*N* = 4824). **f** Same as **e** but for yearly mean values (*N* = 402). **g** Same as **c** but for *N* = 20,000 training months. **h** Same as **d** but using the Last Millennium Reanalysis (LMRv2) as baseline data set (*N* = 397). The location of the pseudo-stations is indicated in Fig. 2h as yellow diamonds. All non-grey fields are significant on a 95% level. Values on the right side of the plot title indicate global mean Pearson correlation coefficient values.

Figure 2 shows the temporal correlation between EKF400v2 and the MPI-GE reconstruction, highlighting regions of weak and strong correlation skill. As expected, correlations for the Southern Hemisphere, where no observations were available, are on average substantially lower than for the Northern Hemisphere.

For tropical regions, near-surface temperatures within the El Ni$\tilde{{{{{{{{\rm{n}}}}}}}}}$ o domain show only weakly positive correlations. We found that all reconstructions, independent of the training data set, struggle in this region. The Eastern Pacific is a region of high temperature variability and a trigger for non-stationary climate tele-connections around the globe. We define tele-connections in our context as a relationship between a variable or variables over large distances. The correlation coefficient values here not only depend on how well these climate tele-connections are reproduced by and copied from MPI-GE, but also on the quality of the EKF400v2 SST boundary conditions. Particularly for the early centuries, the EKF400v2 intra-annual SST variability is represented by climatology values blended with an El Ni$\tilde{{{{{{{{\rm{n}}}}}}}}}$ o regression^42^. A region of pronounced negative correlations manifests over equatorial Western Africa. We find that in this case, those differences are specific to MPI-GE as a training data set and do not occur for 20CRv3 nor CESM-LME. As such, they are a result of data artifacts in MPI-GE (for correlation analysis with reconstructions based on 20CRv3 and CESM-LME see Supplementary Figs. S1 and S2).

For the Northern Hemisphere, the central North Atlantic temperature variability is difficult to reconstruct for the RNN, a feature that occurs for any training data set but is less pronounced for 20CRv3. Assimilated ship-based pressure observations in 20CRv3 might help to overcome this weakness. An interesting region with lower reconstruction skill is the western United States, where all training data sets show reduced performance. We find that EKF400v2 does not have high confidence in this region except for the boreal summer season (JJA) where most of the data in this region are assimilated^24^.

As expected, global average boreal summer correlation coefficients and the associated significance (Fig. 2a) are generally lower than for boreal winter (Fig. 2b). Summer climate patterns are often driven by local, dynamical processes between the surface and the free atmosphere, making them hard to predict via large-scale climate interactions. An example is the Eastern Mediterranean region, which is well covered by assimilated data, but is governed by rather local climate variability, inhibiting reconstruction skill by the MPI-GE trained RNN. Northern Hemisphere winter on the other hand is marked by strong latitudinal temperature differences, producing stronger atmospheric waves which can be used to predict local temperature anomalies. High latitude regions where the near-surface temperature variability is mostly dominated by atmospheric large-circulation anomalies are especially well reconstructed. Surprisingly, the Southern Hemisphere summer variability is also well captured in our reconstruction. This result can be partly explained by the generally lower magnitude of climate anomalies predicted by the RNN, which are far from the pseudo-station locations.

Furthermore, correlating monthly 2m temperature anomalies (Fig. 2c) results in generally lower correlation coefficients than those produced for seasonal means. This is partly explained by the increased sample size. That said, annual mean correlations (Fig. 2d) show high skill across the globe, taking advantage of smoothing intra-annual variability. Figure 2e, f exhibits the impact of increased noise in the time series. Instead of extracting local data from the EKF400v2 ensemble mean, smoothing inter-member differences (or sampling uncertainty), we extracted pseudo-station data from each of the 30 EKF400v2 members, creating 30 noisy MPI-GE-REC realisations. The ensemble mean of those 30 reconstructions is then employed to calculate the correlation with the EKF400v2 ensemble mean. As anticipated, correlation coefficients are overall lower in this case.

The reconstruction skill can be improved by increasing the size of the training data set. Figure 2g shows the impact of increasing the training sample size by a factor of 10. Among the improved regions are the aforementioned Western United States, equatorial Western Africa and the Eastern Mediterranean. Southern Hemisphere regions profit less from prolonged training. Lastly, Fig. 2h investigates the annual correlation skill with the completely independent Last Millennium Reanalysis (LMRv2, available in annual resolution only)^43^. Since LMRv2 uses a different approach than EKF400v2 to reproduce annual climate variability and is completely independent of the pseudo-station data used to generate the reconstruction, global correlation skill is lower, as can be expected (for a comparison between EKF400v2 and LMRv2 directly see Fig. S7). Similar regions appear to be challenging for the RNN reconstruction, such as the Southern Ocean, the El Ni$\tilde{{{{{{{{\rm{n}}}}}}}}}$ o domain, the central North Atlantic and Eastern Mediterranean. However no additional inconsistencies or artifacts appear in this analysis. It is worth mentioning that both baseline data sets, LMRv2 and EKF400v2, are imperfect reconstructions and carry uncertainties into the correlation analysis.

**Fig. 3:**
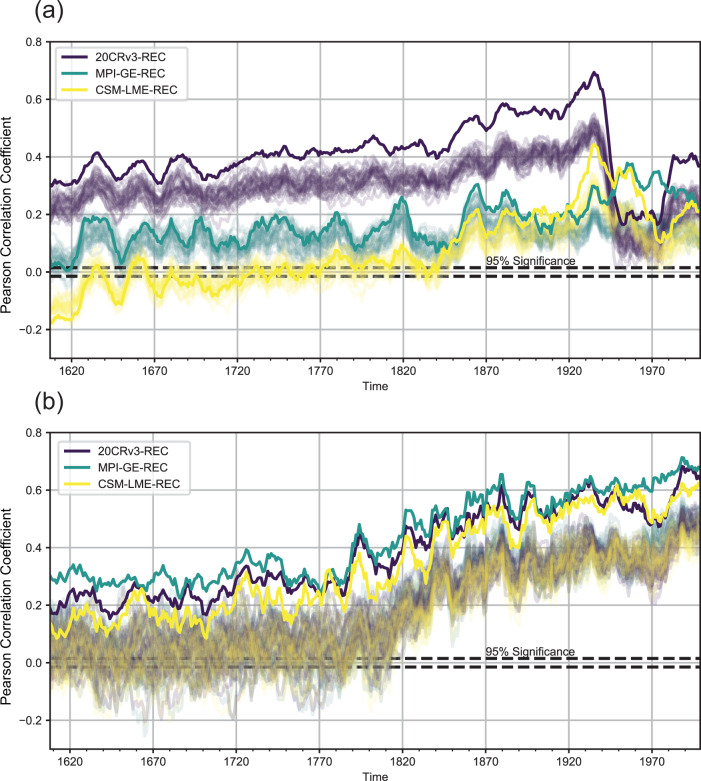
Reconstruction performance over time. 10-year running mean time series of Pearson correlation coefficients between EKF400v2 ensemble mean and the RNN temperature anomaly reconstruction (*N* = 1980 training months) fields per season (each time step contains *N* = 18,432 grid values). Solid lines represent correlations between the EKF400v2 ensemble mean and the reconstruction based on extracting pseudo-station data from the EKF400v2 ensemble mean. The 30 transparent lines represent correlations between the EKF400v2 ensemble mean and the reconstruction based on extracting pseudo-station data from the individual EKF400v2 member. **a** Correlation coefficients for boreal summer (JJA) seasons. **b** Correlation coefficients for boreal winter (DJF) seasons.

To investigate the time-dependent performance of RNN reconstructions, Fig. 3 displays field correlations for boreal summer and winter. Compared to the previous temporal correlation analysis, field correlation uses co-variance in space across the full grid.

Looking at the time series for boreal summer (Fig. 3a), the 20CRv3-REC exhibits a substantially higher correlation skill compared to the MPI-GE-REC and CESM-LME-REC, with MPI-GE-REC outperforming CESM-LME-REC for most decades. This can be explained by the more realistic depiction of summer climate in 20CRv3 (and EKF400v2) due to assimilation of surface pressure data. The CESM-LME-REC shows negative or insignificant correlation coefficients for two thirds of the reconstructed era. This behaviour could be due to a substantial weakness of CESM-LME in reproducing JJA climate patterns, the lower spatial resolution of the original product, or the fact that CESM-LME covers a much wider range of climate background states (going back to 850 CE), from which we randomly sample only 1980 months. As such, it is possible that we sampled climate conditions that are very different to the ones in the period 1602-2003 during the training of the LSTM. However, decadal variability and a general increase in correlation skill over time is visible in all three reconstructions. Decadal variability is determined by how well the three climate data sets reproduce temperature impacts by events such as volcanic eruptions or El Ni$\tilde{{{{{{{{\rm{n}}}}}}}}}$ o - Southern Oscillation. Generally, the reconstructions based on ensemble mean pseudo-stations show higher correlation than the noisier input data. This difference increases with time due to higher certainty in the EKF400v2 ensemble.

The positive trend in correlation skill over time is mostly governed by better representation of climate patterns with time in EKF400v2 as well as more familiar climate background conditions for the training data sets (with MPI-GE and 20CRv3 starting in 1851). The remarkable drop of skill for the 20CRv3-REC in the 20th century is an artifact due to a decrease of global mean co-variance in both, 20CRv3-REC and EKF400v2. Using anomalies with respect to 1951–1980 reduces the spatial co-variance in both ensemble mean products for this time period, resulting in reduced correlation skill. This feature is less prevalent in the model-based reconstructions since we computed anomalies with respect to an all-member average climatology, leaving individual members with a higher co-variance for that time period. We speculate that this feature appears only in JJA due to the higher spatial climate variability in individual December–January–February (DJF) seasons.

In winter, EKF400v2 assimilates less data than in summer. Nevertheless, like all training data sets, EKF400v2 winter climate is more influenced by planetary wave interactions which act as a source of skill for inter-continental climate reconstructions. This leads to all three reconstructions showing very similar overall skill, decadal variability and improvement over time (Fig. 3b) for boreal winter patterns. Therefore all three training data sets provide a similar picture of DJF near-surface temperature variability, with EKF400v2 itself being more model-like. Here, MPI-GE-REC even outperforms the 20CRv3-REC consistently over all decades. This is a testament to how well MPI-GE manages to represent boreal winter climate tele-connections. The improvement of correlation skill over time can most likely be attributed to the incorporation of instrumental data in EKF400v2 (rather than warm season proxies and documentary data) as well as to the aforementioned familiarity of the training data sets with the reconstructed era.

Performing the same field correlation analysis using the independent period of 1836–1850, and comparing to the 20CRv3 ensemble mean rather than to EKF400v2, we see generally the same behaviour as in Fig. 3, with our 20CRv3-REC outperforming EKF400v2. During DJF, all reconstructions show equal performance (see Supplementary Fig. S3). That said, 20CRv3 in these early periods can be assumed to be highly uncertain.

Finally, we evaluate reconstructed climate variability by checking for the leading principal components of 2m temperature anomalies in the global reconstructions. We find realistic first and second rank empirical orthogonal functions (EOFs) for monthly 2m temperature anomalies in all reconstructions compared to EKF400v2 (see Supplementary Fig. S4). The 20CRv3-REC EOFs show the closest resemblance to EKF400v2 due to improved representation of summer near-surface temperature variability (Fig. 3a). Increasing the amount of observations would naturally improve the representation of EOFs, especially for the Southern Hemisphere.

We can thus argue, that, given that the training data set represents all features of a realistic temperature variability, the neural network is able to catch and represent those features accordingly.

### Mean biases of reconstructed fields

**Fig. 4:**
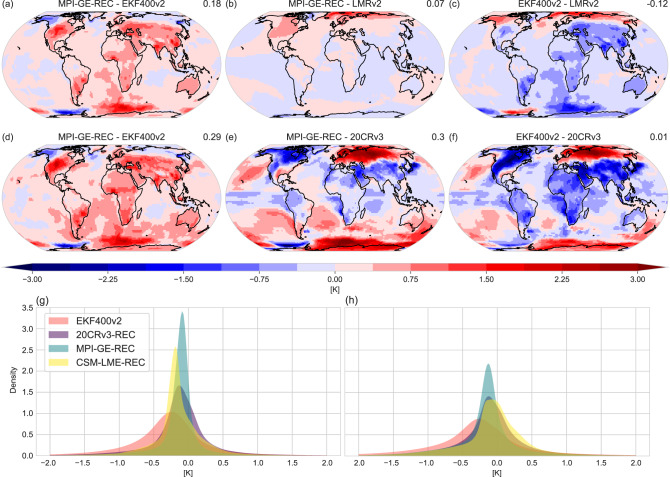
Mean differences between reconstructed and reanalysed 2m temperature anomalies. **a** Mean differences between MPI-GE-REC and EKF400v2 for all time steps 1602–2003 CE. **b** Mean differences between MPI-GE-REC and LMRv2 for all time steps 1602–2000. **c** Mean differences between EKF400v2 and LMRv2 for all time steps 1602–2000 CE. **d** Mean differences between MPI-GE-REC and EKF400v2 for all time steps 1836–1850. **e** Mean differences between MPI-GE-REC and 20CRv3 for all time steps 1836–1850 CE. **f** Mean differences between EKF400v2 and 20CRv3 for all time steps 1836–1850 CE. **g** Kernel density distribution for all monthly values and all grids in EKF400v2 and three RNN-based reconstructions. Distribution is cut off at −2 and 2 Kelvin. **h** Same as **g** but for Northern Hemisphere grids only (30–90^∘^ N). Values on the right side of the plot title above maps indicate global mean values.

Assessing temperature magnitude biases depends on the training data set, the amount of stations and the imperfections in the base line data set. Figure 4 investigates temperature biases for two time intervals, an overall assessment for the reconstructed 400 years and a shorter time period of 15 years (1836–1850) in order to compare to 20CRv3. MPI-GE-REC shows wide-spread positive temperature biases for most of the continental and marine grid points with respect to EKF400v2 (Fig. 4a). Strongest biases occur over the Southern Ocean, likely as a result of the differences between the coupled ocean in MPI-GE and the reconstructed SSTs in EKF400v2. Arctic regions are slightly cooler in the MPI-GE-REC, a region of little to no assimilation in EKF400v2. On the other hand, the comparison to LMRv2 highlights the Arctic as the main region with positive temperature biases in the MPI-GE-REC (Fig. 4b). As such, EKF400v2 itself might be biased towards too warm temperature anomalies in the Arctic. Globally, the comparison with LMRv2 shows reduced biases for all regions outside of the Arctic (Fig. 4c). Comparing both paleo-reanalyses reveals strong differences in the Southern Ocean and higher temperature anomalies in LMRv2 globally, except for the Arctic.

Analysing a much shorter period in the 19^th^ century reveals similar biases between EKF400v2 and the MPI-GE-REC (Fig. 4d): positive temperature biases globally except over the Arctic. This pattern changes when compared to 20CRv3, apart from mismatches in the Southern Ocean. Strong positive temperature biases occur over Northern Eurasia, with strong negative biases over large parts of North America. Meanwhile weak negative temperature biases dominate most of the remaining continents (Fig. 4e). Those discrepancies are generally amplified in the comparison between EKF400v2 and 20CRv3 (Fig. 4f). These results support the notion that EKF400v2 might be too warm in the European Arctic and too cold for many continental regions. It is not surprising that Fig. 4e, f shows generally similar patterns, since the MPI-GE-REC uses pseudo-stations based on EKF400v2 grid values (spatial biases for the 20CRv3-REC and CESM-LME-REC reconstructions are shown in Supplementary Figs. S5 and S12)

To examine the impact of the different training data sets on the temperature bias, Fig. 4g displays the Kernel density distribution of all temperature values over all grids and months for EKF400v2 and different RNN reconstructions. Choosing a reference period in the second half of the 20th century results in the distribution median for the whole time period being located in the slightly negative temperature anomaly range. In general, the RNN reconstructions show a narrower distribution with less extreme values. This behaviour is expected when comparing few pseudo-stations to a product assimilating much more data. Interestingly, the 20CRv3-REC achieves a wider distribution compared to the model-based reconstructions, probably due to better representation of summer temperature patterns. Another feature present in Fig. 4a, is the overall shift of the distribution towards more positive temperature values in the RNN reconstructions. This might reflect the challenges of the RNN reconstructions in capturing cold extremes, but could also be a result of biases in EKF400v2 (Fig. 4c, f).

Focusing on the Northern Hemisphere, the distribution of RNN reconstructed temperature anomalies become more aligned with EKF400v2 (Fig. 4h). Interestingly, the MPI-GE-REC’s median is now closest to EKF400v2, yet still undersampling extreme values. Nevertheless, the positive temperature bias in the RNN reconstructions persist for the Northern Hemisphere sub-sample. Overall, the RNN reconstructions are able to reproduce a realistic distribution, where the exact shape and median depend on the training data sets and the number of pseudo-stations.

So far we have analysed the overall statistical performance of the MPI-GE-REC. However, climate reconstructions are often used to investigate case studies and as such should reproduce individual events in the correct magnitude and location. In order to have good amount of independent data to compare to, we analysed several case studies from the early 19th century cold seasons (Oct–May) (See Supplementary Figs. S8–S12). As independent data sets we utilize (an experimental version of) 20CRv3, EKF400v2 and a recently released Bayesian cold season reconstruction for the 19th century (CSR)^16^ in order to compare temperature anomalies to MPI-GE-REC. We find that 20CRv3 is often times the outlier between all four data sets, with the least sophisticated MPI-GE-REC providing reasonably similar maxima and minima locations as EKF400v2 and CSR. In general, MPI-GE-REC shows a slightly reduced anomaly magnitude compared to the other three products. This behaviour is a result of the temperature anomaly magnitudes represented in the training data sets, as well as the lower magnitudes of the pseudo-station anomalies compared to the magnitude of the assimilated data in the reanalyses. Still, the magnitude of anomalies depends on the setup of the RNN and can be improved with different input and training data sets. Note that due to the temporal limitations of the CSR, we are forced to use a different reference period. Thus CSR anomalies should mostly be seen as qualitative check for the location of anomalies, since we would expect positive anomalies to be weaker and negative anomalies to be stronger when compared to 1951–1980.

### Linear versus non-linear reconstructions

**Fig. 5:**
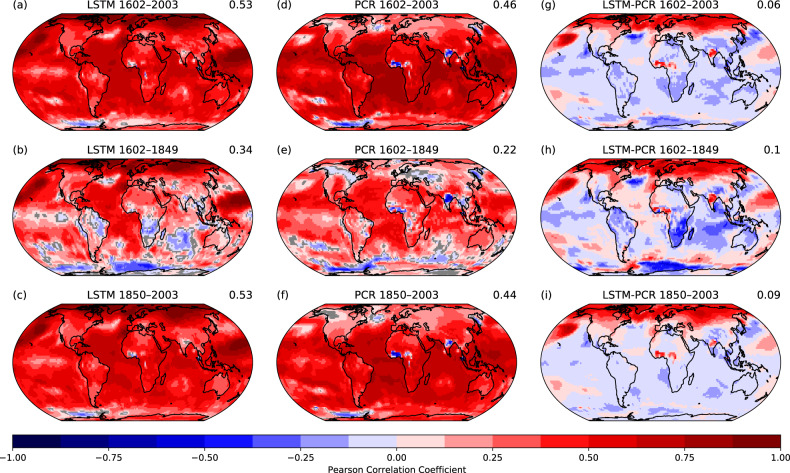
Performance compared to established reconstruction method. Maps of Pearson correlation coefficients between EKF400v2 ensemble mean and the LSTM trained on MPI-GE (MPI-GE-REC) as well as the PCR trained on MPI-GE (*N* = 20,000 training months) monthly temperature anomaly reconstruction for the period 1602–2003 CE. **a** Correlation coefficients between monthly temperature anomalies reconstructed by the LSTM trained on MPI-GE (MPI-GE-REC) and EKF400v2 ensemble for the period 1602–2003 CE. **b** Same as **a** but for 1602–1849 CE. **c** Same as **a** but for 1850–2003 CE. **d** Correlation coefficients between monthly temperature anomalies reconstructed by the PCR trained on MPI-GE (MPI-GE-REC) and EKF400v2 ensemble for the period 1602–2003 CE. **e** Same as **a** but for 1602–1849 CE. **f** Same as **a** but for 1850–2003 CE. **g** Correlation coefficients anomalies (**a**) minus (**d**). **h** Correlation coefficients anomalies between (**b**) minus (**e**). **i** Correlation coefficients anomalies (**c**) minus (**f**). Values on the right side of the plot title indicate global mean values.

In order to assess the performance of MPI-GE-REC compared to a PCR reconstruction, Fig. 5 shows correlation coefficients between the two reconstructions and EKF400v2 temperature anomalies. Since the PCR reconstruction requires a calibration period, we split Fig. 5 in three time slices, (1) the complete time period 1602–2003 CE, (2) the pre-calibration period 1602–1849 CE and (3) the calibration period 1850–2003 CE (which is the overlap of the time span covered by MPI-GE and EKF400v2). Note that we expect the PCR to benefit greatly from this bias-reduction procedure, which the LSTM can not access. We show this performance assessment for the MPI-GE training data set only, due to time and space constraints. For all three time periods, we see a slightly better performance of the LSTM on a global level. Interestingly, the strongest difference in performance can be found for the pre-calibration period, possibly due to more flexibility and non-linearity in the neural network. That said, striking regional differences appear. The deep learning reconstruction substantially outperformed the PCR reconstruction in the northern extra-tropics. The PCR reconstruction on the other hand shows a better performance over the tropics and parts of the extra-tropical oceans. While MPI-GE artifacts have stronger negative impacts for the PCR reconstruction, the Southern Ocean is better represented by these linear models. This seems to hint towards an easier, systematic bias in the SSTs that can be reduced by learning from the calibration method, whereas the artifacts over western Africa and Northern India are less systematic.

We explain the strong performance of the deep learning based reconstruction due to its ability to assess evolution from month to month, from location to location as well as its ability to reconstruct better non-linear features. The atmospheric dynamics in the Northern Extra-tropics are driven by large planetary waves which move information and energy across longitudes and in time. This time dependency is better caught by the LSTM as well as its ability to extrapolate to new atmospheric conditions from training to prediction. Moreover, the month-to-month signal propagation is much stronger in the extra-tropics than the tropics, where the seasonality is substantially weaker.

Concerning the strong PCR performance in the tropics, we assume that the calibration between the MPI-GE ocean and the EKF400v2 SSTs allows to catch the “right” (right as in close to the target, in our case EKF400v2) climate tele-connections, tropical features and to remove over- or underrepresentation of climate signals. Moreover, signal transport in oceans can be a magnitude slower than in the atmosphere, meaning there would probably need to implement multiple time scales in the training procedure to reconstruct oceans and atmosphere in the best way possible. On the other hand, tropical climate over land is mostly defined by daily climate, which the LSTM can not learn from monthly anomalies.

Nevertheless, the global performance can of course be improved with different pseudo-station locations. Due to the nature of the method, the PCR is allowed to get a glimpse into the test data set (something not allowed for the models based on neural networks), which in turn is constrained to reality by assimilating real-world data. We find that the LSTM is a great addition to the PCR, especially skilled for the reconstruction of extra-tropical regions.

## Conclusion

With the recent convergence of open-access big-data availability and user friendly, easy-to-implement machine learning software packages, there is a huge untapped potential for providing non-linear, flexible climate field reconstructions and services to the community. By utilising existing data, storage and energy consumption can be kept at a minimum, while at the same time contributing to the United Nations Sustainable Development Goals.

To prepare for the challenges of the 21st century, equalizing access to and production of knowledge needs to be a key goal for the upcoming decades. Our approach shows that machine learning generated knowledge can help decentralise climate expertise. The RNN reconstructions can be created in less than an hour on a average-priced laptop, operating solely with open-access, open-source software and data. Adding GPU access will increase the speed of the reconstruction, but is not crucial.

We focus here on a grid reconstruction problem, a typical issue in the (paleo-) climate community. By using a very conservative approach with small sample sizes (especially for deep learning), we could produce a realistic, robust global temperature reconstruction. Compared to previous gridded climate reconstruction techniques^31,32^, we use sparse time series to reconstruct a grid of temperatures with four orders of magnitude more data. However, our methodology can be improved in a few ways: (a) more data can be incorporated; (b) meta-heuristic algorithms can be applied to find optimal measuring locations; (c) seasonal information and co-variate data can be incorporated as an additional layer; (d) longer training periods can be performed; (e) hyper-parameters and loss function can be tuned more vigorously and (f) larger ensembles of reconstruction can be computed.

In terms of training data set selection, we could see that data assimilation in 20CRv3 helps reconstructing summer features correctly. This comes with the drawback of smaller training sizes. In the end, it is up to the user to decide which features are most important for the reconstruction and should thus decide on the training data set. In our case, we would refrain from recommending CESM-LME for reconstructing EKF400v2, since it shows apparent flaws compared to 20CRv3 and MPI-GE.

On the other hand, additional challenges could occur when modifying our method. 2m temperatures are one of the easiest climate variables to reconstruct. Meanwhile, reconstructing short-term, local processes such as precipitation, will be more challenging for the RNN to learn, while large-scale, long-term processes like SSTs will be easier to reconstruct. In-situ data like proxy or station data will be less homogeneous, less complete, and noisier than our pseudo-station data. We tried to evaluate the impact of additional noise for the input data and found an expected decrease in reconstruction skill. To counter-act the impact of noise, we find that training with noisy data such as using 20CRv3 individual members rather than ensemble mean, and then creating large ensembles of reconstructions is a good solution (not shown). Moreover, we find that both, long-term variability patterns expressed in EOFs as well as specific, single-season case study temperature anomaly patterns can be reconstructed accordingly by a RNN.

We see potential for this approach to reconstruct climate variables from (paleo) climate archives such as tree rings, coral and ice cores, even without forward-modelling temperature from the proxy in the first place. Future studies will investigate different types of input data in time and space, with varying uncertainty and resolution. Moreover, with novel deep learning architectures, non-numeric historical documents could also be used as input information, reconstructing climate fields from text and image data sets. Needless to say, reconstructions do not need to be global, but can focus on a region of interest. It is also possible to reconstruct multiple variables from single variable input data. In the context of paleo-reanalyses, spatial RNN reconstructions of specific variables or regions could then be assimilated, reducing the uncertainty of the reanalysis outcome. With paleo-reanalyses performing best during warm seasons, the tested PCR performing best over the tropics and our promising RNN-performing best for boreal winter and the extra-tropics, all three spatial reconstruction methods complement each other excellently.

## Methods

### Neural Network Architecture

Overall we investigated 20 different models using three different data sets and two different training sizes for this study. The models used were the simple Recurrent Neural Network (RNN), Long-short Term Memory models (LSTM), Gated Recurrent Unit models (GRU) and 1-dimensional Convolutional Neural Networks (CNN). Each of these models were then executed with a variety of dropout and layer architectures. Summed up over all these different contributors, we analysed 140 different deep learning models for this climate reconstruction task (a list of those models can be found in the Code Availability git repository). After evaluation of those models, we decided to focus one long short-term memory (LSTM) recurrent neural network with 955,232 trainable parameters, one layer of 64 neurons and 5% dropout. A LSTM is an archetype of neural network designed to memorize sequences of data for multiple time steps^44^. Through a series of feedback connections, the LSTM processes the input information sequentially, storing the previous hidden state through time. This makes it an excellent architecture to reconstruct the climate of the past using observational and proxy time series. In our case, we utilized a small LSTM with an output dimensionality of 64 neurons and the hyperbolic tangent as activation function, followed by a dense layer of 18,432 parameters that were reshaped into a grid of 96 × 192 temperature points. The neural network was trained with three features (i.e., latitude, longitude, and 2m temperature anomaly) using the ADAM optimizer^45^ with a learning rate of 10^−4^ and the Mean Squared Error as loss function. Five ensemble members were then generated and subsequently averaged for each output of the neural network, creating a total of 4824 reconstructed months of 2m mean temperature anomalies. Note that, the training and validation data sets were composed of 80% and 20% of the available information, respectively. With this setup, the training process in a middle-class laptop takes less than an hour to complete on CPU, and five to ten minutes on a standard GPU (e.g., NVIDIA GeForce RTX 3050 Ti).

### Selection of pseudo-station data

To pick realistic locations for possible temperature records, we use the International Surface Temperature Initiative (ISTI) data bank^46^ and choose 25 stations with records that span continuously multiple centuries and thus go back into the 19th or 18th century (Table 1). As this study represents a proof-of-concept, the absolute amount of locations is not crucial for the reconstruction procedure. That said, the more locations available, the better the reconstruction. Our example selection was chosen based on a semi-realistic distribution of historical temperature data in time and space. This conservative approach means that all of our station locations are in the Northern Hemisphere, with most of them located in either Europe or North America. Note that these locations are by no means sampling the best possible co-variability in space for 2m temperatures, especially so with many stations being clustered together in Central Europe. As such, not only more data, but also optimal data locations would improve the results.

**Table 1:** Selection of long-term ISTI stations used for sampling pseudo-station locations in EKF400v2.

Station ID	Longitude	Latitude	Start year	End year
USC00508503	−135.33	57.05	1828	1990
USC00226177	−91.34	31.59	1799	2005
USW00013880	−80.04	32.90	1823	2005
USW00014735	−73.81	42.74	1795	2005
IN020040900	80.18	13	1796	2005
IN024140300	88.33	22.53	1816	2005
RSM00022550	40.73	64.5	1813	2005
RSM00024959	129.72	62.02	1829	2005
RSM00030710	104.35	52.27	1820	2005
RSXLT181644	37.56	55.75	1779	2005
SNXLT983389	103.9	1.3	1825	1984
AU000005010	14.13	48.05	1767	2005
BE000006447	4.35	50.8	1794	2005
FIE00142246	25	60.13	1829	2005
FRM00007150	2.45	48.97	1757	1995
GME00121150	13.31	52.56	1701	2005
ITE00105250	13.35	38.11	1791	2005
LH000026730	25.1	54.63	1777	2005
NO000098550	31.1	70.37	1829	2005
NOE00111040	10.45	63.41	1761	2005
UKXLT476306	−3.35	55.95	1764	2005
UKXLT793735	0	51.5	1763	1969
UKXLT912439	−3.1	57.6	1781	1975
UPM00033345	30.53	50.4	1812	2005
USW00012836	−81.75	24.55	1830	2005

As the selected ISTI stations have missing data and other inhomogeneities in their records, we sub-sample EKF400v2 2m temperature values with a nearest-neighbour approach at the respective ISTI locations. This approach allows us to have 25 continuous, homogeneous 2m temperature time series. That said, this approach also smoothes the temperature record, since the temperature from a model grid represent average temperature over a larger region. We try to assess the impact of smoothing for the reconstruction procedure by looking at individual ensemble member in EKF400v2 (see below).

### Paleo-reanalyses and cold season reconstruction

As a source data set for the pseudo-station data as well as a benchmark data set for global 2m temperature anomalies, we use the updated global atmospheric paleo-reanalysis covering the last 400 years (EKF400v2)^24^. EKF400v2 is based on the ECHAM5.4 global circulation model in atmospheric-only configuration with reconstructed sea surface temperatures to provide a fully global, monthly reanalysis for 1602–2003 CE. It consists of 30 ensemble members and assimilates instrumental, documentary and proxy data using an offline Ensemble Kalman Filter approach. EKF400v2 was found to perform well when compared to independent records^24^. We downloaded all 30 individual member for monthly 2m temperature fields on a T63 resolution from https://cera-www.dkrz.de/WDCC/ui/cerasearch/entry?acronym=EKF400v2.0. We calculate monthly, member-specific 2m temperature anomalies with respect to the monthly climatology of 1951–1980 CE. We use nearest-neighbour selection to get the pseudo-station data from the EKF400v2 grids, resulting in 25 time series with *N* = 4824 months to reconstruct. To investigate the impact of uncertainties in the data, we use individual member as well as ensemble mean pseudo-proxy data for the neural network reconstruction.

For an independent evaluation of annual 2m temperature anomalies, we use the updated Last Millenium Reanalysis (LMRv2)^43^. LMRv2 also uses an offline Ensemble Kalman Filter approach, but key differences exist compared to EKF400v2. Firstly, LMRv2 only assimilates proxy data, rather than instrumental or documentary data. It further uses the CCSM4 Last Millennium simulation as the source of prior, where the prior is generated by randomly sampling 100 randomly drawn years. LMRv2 covers the time period 0–2000 CE, and we use an ensemble mean of the years 1602-2000 for comparison with EKF400v2 and the neural network reconstruction. We downloaded the LMRv2.1 data on T42 resolution from https://atmos.washington.edu/~hakim/lmr/LMRv2/. LMRv2 2m temperature data is only available as anomalies with respect to the 1951–1980 CE climatology.

For an independent evaluation of cold season 2m temperature anomalies, we use a newly published climate field reconstruction for the years 1701–1905 (CSR)^16^. The October–May average temperature field reconstructions were computed using a Bayesian reweighting method with the same ECHAM5.4 model run of EKF400v2 as prior, however using completely independent, newly digitized plant and ice phenology data to constrain the weights of each field. We downloaded the CSR data on T63 resolution from 10.1594/PANGAEA.934288.

### Training data

For training we investigate three different gridded climate data sets; one climate reanalysis and two coupled general circulation models.

For reanalysis data, we use the National Oceanic and Atmospheric Administration 20th Century Reanalysis Version 3 (20CRv3)^22^. Monthly mean, ensemble mean (of 80 ensemble members) 2m temperature data for 1836–2015 CE on a 1 degree spatial resolution were downloaded from https://psl.noaa.gov/data/gridded/data.20thC_ReanV3.html. This reanalysis assimilates surface pressure observations only, with sea surface temperature and sea ice reconstructions as boundary conditions. We use the years 1851–2015 CE (1980 months) for training purposes and leave out the years 1836–1850 for independent evaluation. 20CRv3 was found to represent global climate variability in terms of magnitude, timing and spatial precision, with substantial improvements over previous versions^47^. 2m temperature is among the most skilful variables in the data set, and as such we assume a realistic representation of near surface temperature inter-continental correlations for the neural network to learn.

The second data set we use for training purposes is the Max Planck Institute for Meteorology Grand Ensemble (MPI-GE)^37^. MPI-GE consists of 100 members of the well-tested MPI-ESM1.1^48^ model, run on four different forcing scenarios, including a historical forcing (1850–2005 CE) as well as RCP2.6, RCP4.5, and RCP8.5 (2006–2099 CE). We only use the historical forcing ensemble data on a monthly resolution and download 2m temperature data on a T63 resolution from the DKRZ ESGF-CoG Node https://esgf-data.dkrz.de/search/mpi-ge/. MPI-GE was found to capture temperature variability skillfully^49^. The total amount of available months for training in the historical forcing setup is *N* = 187,200. Out of this data pool, we randomly sub-sample the data to either 1980 or 20,000 months for our training routine.

The third data set we use for training purposes is the National Center for Atmospheric Research Community Earth System Model Last Millennium Ensemble (CESM-LME)^38^. This data set consists of 36 last millennium simulations for 850–2005 CE from NCAR’s *CESM* − *CAM*5_*C*_*N* general circulation model, 13 members including transient forcings (solar radiation, volcanic aerosols, greenhouse gases, land use/land cover conditions and orbital parameters). We downloaded monthly 2m temperature fields on a 1.9^∘^ × 2.5^∘^ spatial resolution for the 851-2005 CE period of the full-forcing CESM-LME at https://www.cesm.ucar.edu/projects/community-projects/LME/data-sets.html. This data set has been successfully used in a variety of studies trying to disentangle the contribution of internal and external climate variabilities^50^. The total amount of available months for training in this forcing setup is *N* = 180,024. Out of this data pool, we randomly sub-sample the data to either 1980 or 20,000 months for our training routine.

For all training data sets, we calculate monthly 2m temperature anomalies with respect to the monthly climatology of 1951–1980 CE. We use bilinear interpolation to regrid all training data to the EKF400v2 grid, namely T63 with 1.875^∘^ spatial resolution. We also utilize two-sided *t*-tests to assess the statistical significance of results shown in Figs. 2 and 3.

### Supplementary information

Supplementary Information

## Data Availability

All gridded data products as well as the ISTI database are freely available for research and education purposes. EKF400v2 can be downloaded at https://cera-www.dkrz.de/WDCC/ui/cerasearch/entry?acronym=EKF400_v2.0. 20CRv3 ensemble mean can be downloaded at https://psl.noaa.gov/data/gridded/data.20thC_ReanV3.html. MPI-GE can be downloaded at https://esgf-data.dkrz.de/search/mpi-ge/. CESM-LME can be downloaded at https://www.cesm.ucar.edu/projects/community-projects/LME/data-sets.html. The cold season reconstruction can be downloaded at 10.1594/PANGAEA.934288.

## Code availability

Code to reproduce the results in this publication can be found at https://github.com/martin-wegmann/RNN_climate_reconstruction.
