# Supplementary material for: Artificial intelligence achieves easy-to-adapt nonlinear global temperature reconstructions using minimal local data
Source: Commun Earth Environ. 2023 Jun 16;4(1):217. doi: 10.1038/s43247-023-00872-9 (PMC11041659; doi:10.1038/s43247-023-00872-9)
Supplement: Supplementary file 1 — Supplementary Information [file 43247_2023_872_MOESM1_ESM.pdf]

Supplementary information.

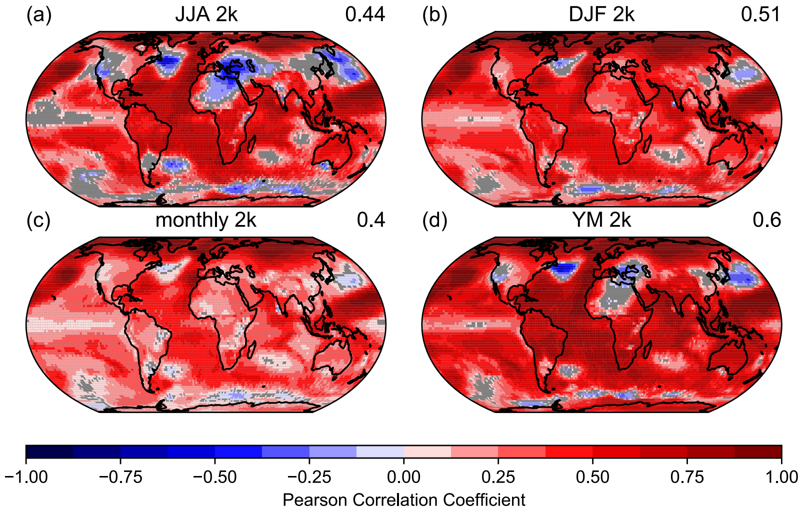

Fig. S 1 Maps of Pearson Correlation Coefficients between EKF400v2 ensemble mean and the CESM-LME-REC (N=1980 training months) temperature anomaly reconstruction for the period 1602–2003 CE. a) Correlation Coefficients for boreal summer (JJA) seasons (N=402). b) Correlation Coefficients for boreal winter (DJF) seasons (N=401). c) Correlation Coefficients for all months (N=4,824). d) Correlation Coefficients for yearly means (N=402). All non-gray fields are significant on a 95% level. Values on the right side of the plot title indicate global mean values.

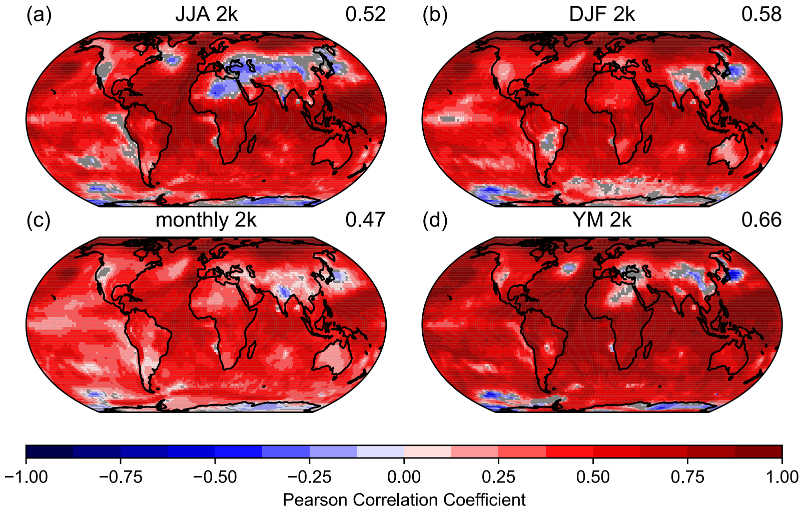

Fig. S 2 Maps of Pearson Correlation Coefficients between EKF400v2 ensemble mean and the 20CRv3-REC (N=1980 training months) temperature anomaly reconstruction for the period 1602–2003 CE. a) Correlation Coefficients for boreal summer (JJA) seasons (N=402). b) Correlation Coefficients for boreal winter (DJF) seasons (N=401). c) Correlation Coefficients for all months (N=4824). d) Correlation Coefficients for yearly means (N=402). All non-gray fields are significant on a 95% level. Values on the right side of the plot title indicate global mean values.

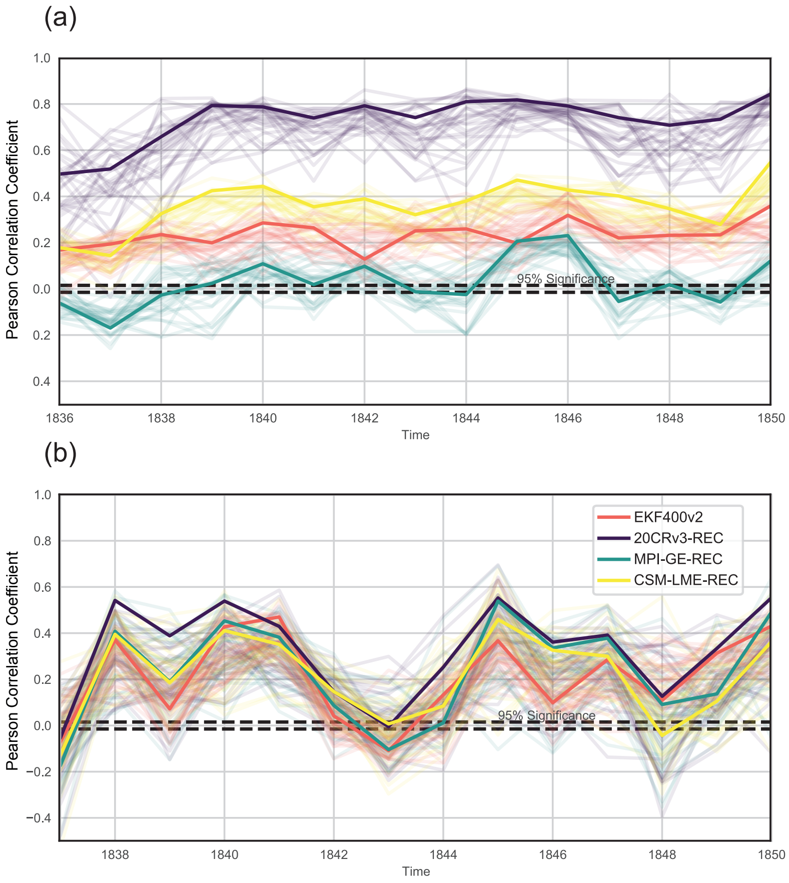

Fig. S 3 Time series of Pearson Correlation Coefficients between 20CRv3 ensemble mean, the EKF400v2 member and the RNN temperature anomaly reconstruction (N=1980 training months) fields per season (each time step contains N=18432 grid values). Solid lines represent correlations between the 20CRv3 ensemble mean, EKF400v2 ensemble mean and the reconstruction based on extracting pseudo-station data from the EKF400v2 ensemble mean. The 30 transparent lines represent correlations between the 20CRv3 ensemble mean, EKF400v2 member and the reconstruction based on extracting pseudo-station data from the individual EKF400v2 member. a) Correlation Coefficients for boreal summer (JJA) seasons. b) Correlation Coefficients for boreal winter (DJF) seasons.

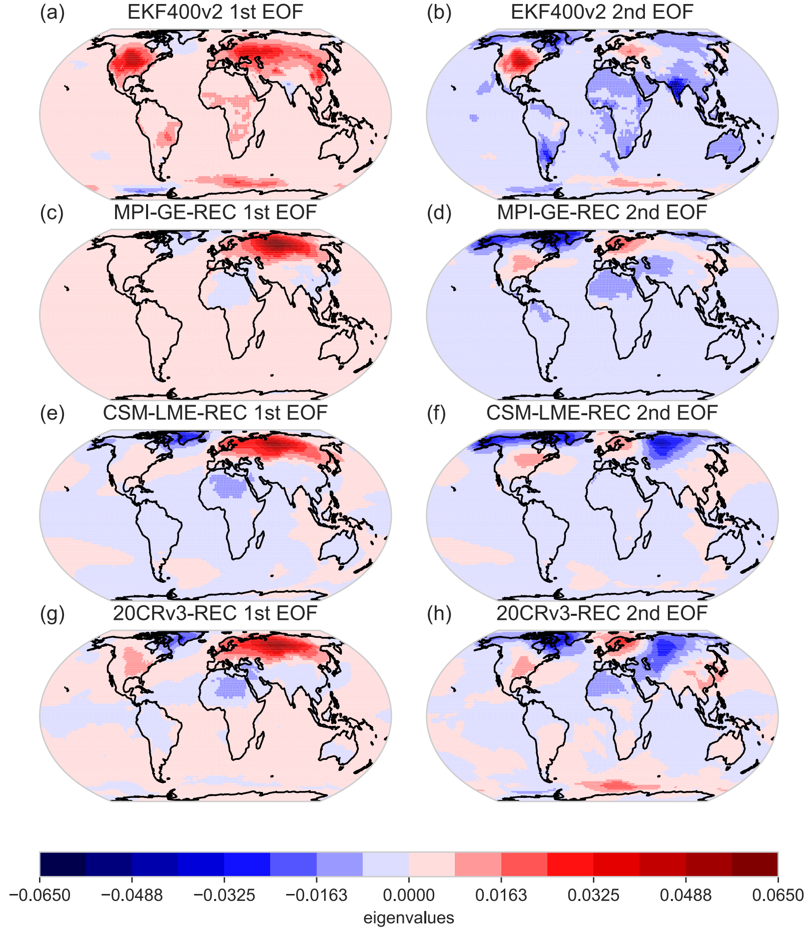

Fig. S 4 First and second EOF loading pattern for monthly 2m temperatures (N=4824). a) First EOF of EKF400v2 reanalysed values. b) Second EOF of EKF400v2 reanalysed values. c) First EOF of MPI-GE-REC values. d) Second EOF of MPI-GE-REC values. e) First EOF of CESM-LME-REC values. f) Second EOF of CESM-LME-REC values. g) First EOF of 20CRv3-REC values. h) Second EOF of 20CRv3-REC values.

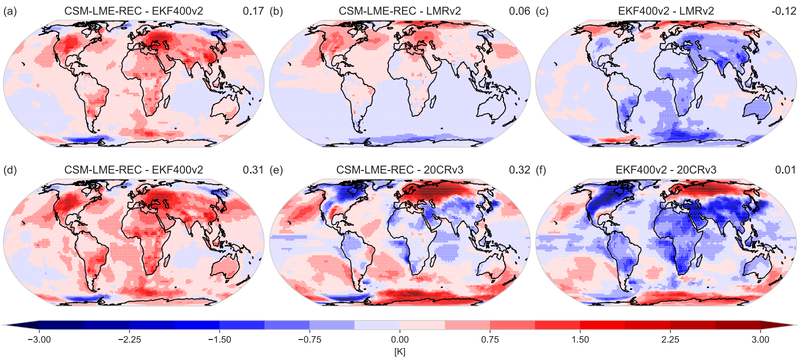

Fig. S 5 Same as Figure 4a-f but for the CESM-LME-REC.

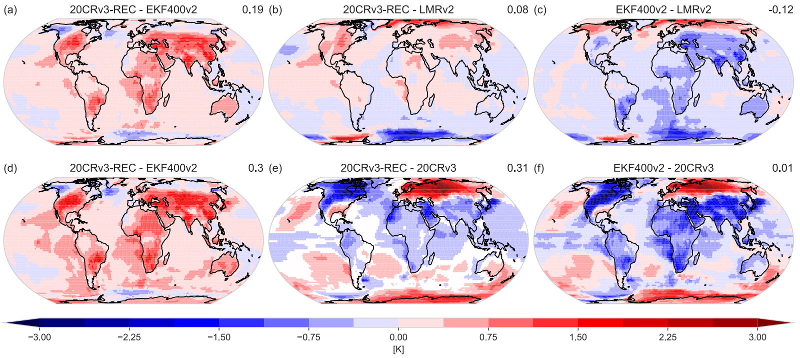

Fig. S 6 Same as Figure 4a-f but for the 20CRv3-REC.

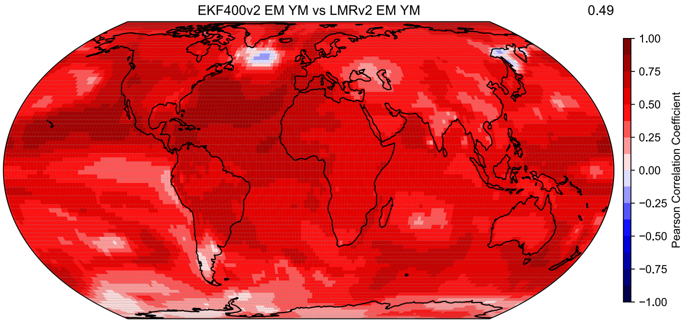

Fig. S 7 Pearson Correlation Coefficient between EKF400v2 and LMRv2 yearly mean, ensemble mean 2m temperature anomalies for the years 1602–2000 CE. Value on the right side of the plot title indicate global mean values.

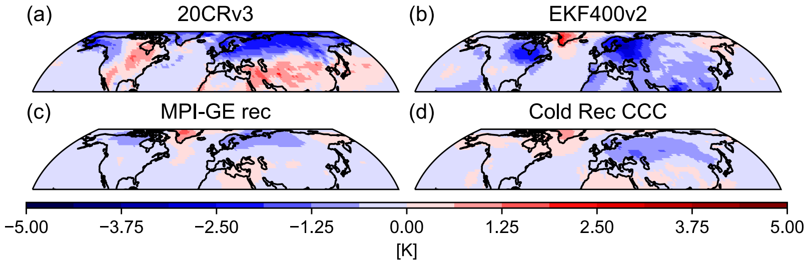

Fig. S 8 Cold season (Oct-May) 2m temperature anomalies for 1808/09 CE. a) Ensemble mean 20CRv3 anomalies with respect to 1951-1980. b) Ensemble mean EKF400v2 anomalies with respect to 1951-1980. c) MPI-GE-REC anomalies with respect to 1951-1980. d) Bayesian cold season reconstruction anomalies with respect to 1851-1900.

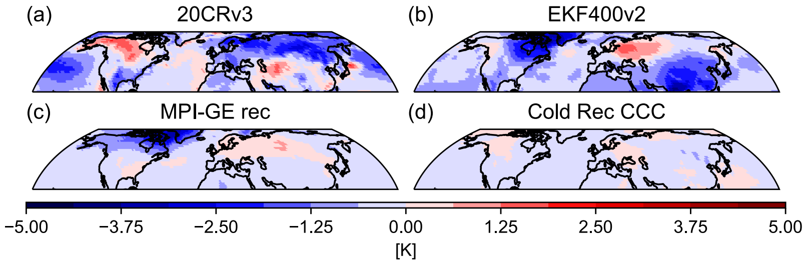

Fig. S 9 Cold season (Oct-May) 2m temperature anomalies for 1816/17 CE. a) Ensemble mean 20CRv3 anomalies with respect to 1951-1980. b) Ensemble mean EKF400v2 anomalies with respect to 1951-1980. c) MPI-GE-REC anomalies with respect to 1951-1980. d) Bayesian cold season reconstruction anomalies with respect to 1851-1900.

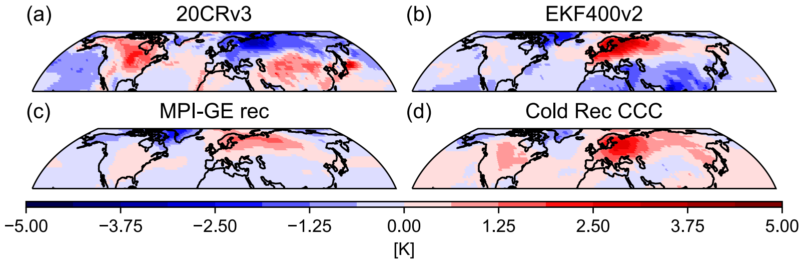

Fig. S 10 Cold season (Oct-May) 2m temperature anomalies for 1821/22 CE. a) Ensemble mean 20CRv3 anomalies with respect to 1951-1980. b) Ensemble mean EKF400v2 anomalies with respect to 1951-1980. c) MPI-GE-REC anomalies with respect to 1951-1980. d) Bayesian cold season reconstruction anomalies with respect to 1851-1900.

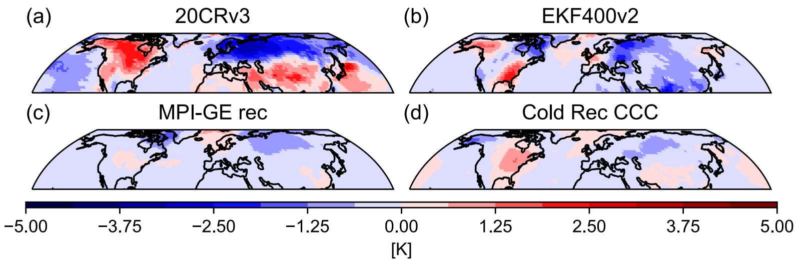

Fig. S 11 Cold season (Oct-May) 2m temperature anomalies for 1827/28 CE. a) Ensemble mean 20CRv3 anomalies with respect to 1951-1980. b) Ensemble mean EKF400v2 anomalies with respect to 1951-1980. c) MPI-GE-based reconstruction anomalies with respect to 1951-1980. d) Bayesian cold season reconstruction anomalies with respect to 1851-1900.

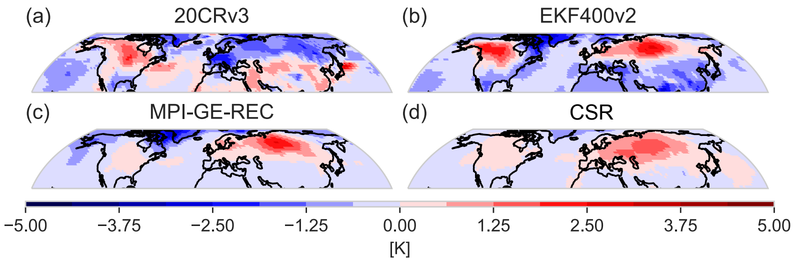

Fig. S 12 Cold season (Oct-May) 2m temperature anomalies for 1834/35 CE. a) Ensemble mean 20CRv3 anomalies with respect to 1951-1980. b) Ensemble mean EKF400v2 anomalies with respect to 1951-1980. c) MPI-GE-REC anomalies with respect to 1951-1980. d) Bayesian cold season reconstruction anomalies with respect to 1851-1900.
